# The impact of radiomics for human papillomavirus status prediction in oropharyngeal cancer: systematic review and radiomics quality score assessment

**DOI:** 10.1007/s00234-022-02959-0

**Published:** 2022-04-23

**Authors:** Gaia Spadarella, Lorenzo Ugga, Giuseppina Calareso, Rossella Villa, Serena D’Aniello, Renato Cuocolo

**Affiliations:** 1 Department of Advanced Biomedical Sciences, University of Naples “Federico II”, Via Sergio Pansini 5, 80131 Naples, Italy; 2 Department of Radiology, Fondazione IRCCS Istituto Nazionale Dei Tumori, Via Giacomo Venezian 1, 20133 Milan, Italy; 3 Department of Clinical Medicine and Surgery, University of Naples “Federico II”, Via Sergio Pansini 5, 80131 Naples, Italy; 4 Interdepartmental Research Center on Management and Innovation in Healthcare—CIRMIS, University of Naples “Federico II”, Via Sergio Pansini 5, 80131 Naples, Italy

**Keywords:** Systematic review, Radiomics, Oropharyngeal neoplasms, Human papillomavirus, Machine learning

## Abstract

**Purpose:**

Human papillomavirus (HPV) status assessment is crucial for decision making in oropharyngeal cancer patients. In last years, several articles have been published investigating the possible role of radiomics in distinguishing HPV-positive from HPV-negative neoplasms. Aim of this review was to perform a systematic quality assessment of radiomic studies published on this topic.

**Methods:**

Radiomics studies on HPV status prediction in oropharyngeal cancer patients were selected. The Radiomic Quality Score (RQS) was assessed by three readers to evaluate their methodological quality. In addition, possible correlations between RQS% and journal type, year of publication, impact factor, and journal rank were investigated.

**Results:**

After the literature search, 19 articles were selected whose RQS median was 33% (range 0–42%). Overall, 16/19 studies included a well-documented imaging protocol, 13/19 demonstrated phenotypic differences, and all were compared with the current gold standard. No study included a public protocol, phantom study, or imaging at multiple time points. More than half (13/19) included feature selection and only 2 were comprehensive of non-radiomic features. Mean RQS was significantly higher in clinical journals.

**Conclusion:**

Radiomics has been proposed for oropharyngeal cancer HPV status assessment, with promising results. However, these are supported by low methodological quality investigations. Further studies with higher methodological quality, appropriate standardization, and greater attention to validation are necessary prior to clinical adoption.

**Supplementary Information:**

The online version contains supplementary material available at 10.1007/s00234-022-02959-0.

## Introduction

Oropharyngeal squamous cell carcinoma (OPSCC) is one of the most frequent head and neck cancer, strictly related to human papillomavirus (HPV) infection in the majority of cases [1]. Despite sharing the same anatomical location, HPV-positive and HPV-negative OPSCCs present crucial differences that must be taken into account by oncologists: 1) clinical presentation, as HPV-positive OPSCC symptoms are related to neck mass due to nodal spread of disease, whereas patients with HPV-negative lesions present symptoms related to local growth of the primary tumour, such as odynophagia and dysphagia; 2) HPV-negative OPSCCs have a lower survival and response rate to radio-chemotherapy than HPV-positive ones; 3) patients affected by HPV-positive OPSCC are often younger than HPV-negative OPSCC [2]. Therefore, HPV status determines the appropriate therapy and follow-up plan. In patients affected by OPSCC, HPV status is routinely assessed on biopsied tissue by p16 immunostaining. However, surgical biopsy exposes patients to surgery-related complications, such as bleeding [3], and the presence of co-existing inflammatory changes in the specimen might decrease the sensitivity of the immunostaining [4].

Despite several studies described different imaging features useful to predict HPV status [5–7], this approach is not sufficiently reliable due to the presence of overlapping radiological characteristics [8]. To overcome the limitations of subjective medical image interpretation, several authors investigated the potential utility of texture analysis in HPV status assessment [9, 10], since one of the aims of radiomics and machine learning (ML) is the conversion of medical images to quantitative, reader independent data for predictive modelling [11, 12].

Radiomics refers to the analysis of large amounts of quantitative features extracted from medical images. These features include pixel grey level distribution parameters and texture analysis derived data, which evaluate grey level value patterns in images. ML is a subfield of artificial intelligence which may be adopted to build up classification or regression models from radiomics data through automated recognition of patterns in the data space, implementing predictive algorithms [11, 13].

Given this potential, recently the number of radiomic studies has grown dramatically, especially in oncological imaging [14, 15]. However, despite these efforts, the routine use of these tools in the clinical setting has not yet occurred, for example due to lack of technique standardization and external validation [14]. The increasing attention given to ML and radiomics has also resulted in a growing availability of quality assessment checklists, such as the Radiomic Quality Score (RQS) [12, 16, 17]. The RQS’s strength is represented by the evaluation of different aspects of radiomic studies, ranging from images acquisition protocol to data sharing, grouped in six domains (protocol quality and reproducibility, feature selection and validation, biologic/clinical validation and utility, model performance index, high level of evidence, and open science and data). Each item contributes to a final percentage score for the paper, allowing for a quantitative assessment of methodological quality. The value of the RQS is also confirmed by its use across various topics in the recent literature [18–20]. An additional advantage of the RQS is the possibility to use its final score to perform statistical analyses with other variables. As also described in a previous report [19], radiomic studies are published on peer-reviewed journals specialized not only in radiology but in a variety of fields, demonstrating a widespread interest among the research community.

With the present systematic review, we aimed to perform a literature revision with RQS quality assessment as well as an evaluation of the relationship between study quality and journal characteristics. In particular, our focus was on the current applications of radiomics in OPSCC imaging for the prediction of HPV status and association between study quality and indices commonly accepted as a proxy for research quality [21].

## Methods

### Article search strategy

**Fig. 1:**
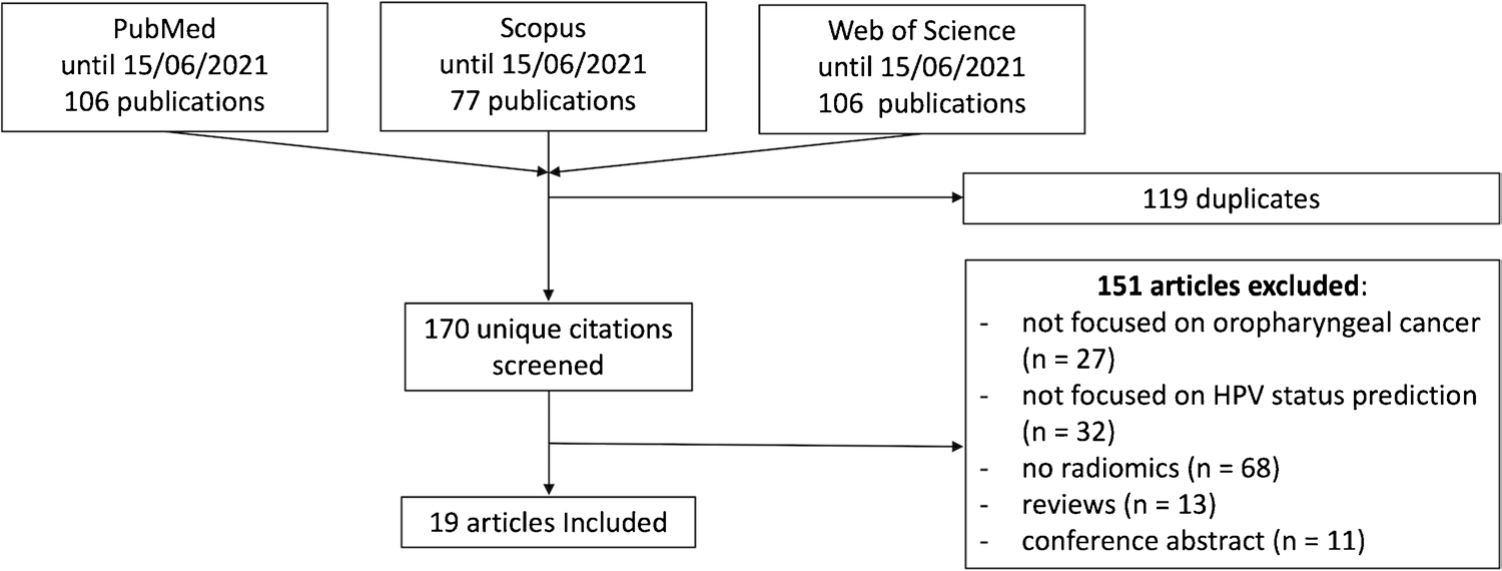
Study selection process flowchart

The selection of included studies was carried out through a detailed search in the field of radiomics in head and neck oncology, conducted according to PRISMA (Preferred Reporting Items for Systematic reviews and Meta-Analyses) guidelines. The study consisted in a systematic search in the electronic databases (PubMed, Web of Science and Scopus) using the following search terms in all possible combinations: radiomics, texture analysis, artificial intelligence, oropharyngeal cancer, Human papillomavirus. The search was finalized on September 1^st^, 2021. Additional details of the literature research are reported in the supplementary materials. As described in Fig. 1, letters, editorials, reviews, duplicates, and articles published in languages other than English were excluded from the analysis.

### Data extraction and analysis

The RQS is a scoring system used to assess the quality of radiomic analysis methodology by assigning a score for each item satisfied in the articles, divided in six domains (image protocol, radiomics features extraction, data analysis and statistics, model validation, clinical validity, and open science). The final score, that ranges from -8 to 36, is then converted to a percentage score (where 36 is equivalent to 100%) [12]. An overview of RQS items and respective scores is provided in Table 1.

**Table 1:** Overview of Radiomic Quality Score items and mode of the respective scores in the reviewed studies

RQS item number and name	Description and (points)
Item 1: Image protocol quality	Well documented protocol (+ 1) AND/OR publicly available protocol (+ 1)
Item 2: Multiple segmentation	Testing feature robustness to segmentation variability: e.g. different physicians/algorithms/software (+ 1)
Item 3: Phantom study	Testing feature robustness to scanner variability: e.g. different vendors/scanners (+ 1)
Item 4: Multiple time points	Testing feature robustness to temporal variability: e.g. organ movement/expansion/shrinkage (+ 1)
Item 5: Feature reduction	Either feature reduction OR adjustment for multiple testing is implemented (+ 3); otherwise (-3)
Item 6: Multivariable analysis	Non-radiomic feature are included in/considered for model building (+ 1)
Item 7: Biological correlates	Detecting and discussing correlation of biology and radiomic features (+ 1)
Item 8: Cut-off analysis	Determining risk groups by either median, pre-defined cut-off or continuous risk variable (+ 1)
Item 9: Discrimination statistics	Discrimination statistic and its statistical significance are reported (+ 1); a resampling technique is also applied (+ 1)
Item 10: Calibration statistics	Calibration statistic and its statistical significance are reported (+ 1); a resampling technique is also applied (+ 1)
Item 11: Prospective design	Prospective validation of a radiomics signature in an appropriate trial (+ 7)
Item 12: Validation	Validation is missing (-5) OR internal validation (+ 2) OR external validation on single dataset from one institute (+ 3) OR external validation on two datasets from two distinct institutes (+ 4) OR validation of a previously published signature (+ 4) validation is based on three or more datasets from distinct institutes (+ 5)
Item 13: Comparison to “gold standard”	Evaluating model’s agreement with/superiority to the current “gold standard” (+ 2)
Item 14: Potential clinical application	Discussing model applicability in a clinical setting (+ 2)
Item 15: Cost-effectiveness analysis	Performing a cost-effectiveness of the clinical application (+ 1)
Item 16: Open science and data	Open source scans (+ 1) AND/OR open source segmentations (+ 1) AND/OR open source code (+ 1) AND/OR open source representative features and segmentations (+ 1)

*RQS* Radiomics Quality Score

The included full-text articles were independently evaluated using the RQS by three raters experienced in artificial intelligence and head and neck cancer (BLINDED: 5 years of experience, BLINDED and BLINDED: 2 years each). Inter-reader intraclass correlation coefficient (ICC) was assessed for both the RQS total and percentage scores, using a two-way, random-effects, single-rater, absolute agreement model.

Furthermore, the included studies were classified based on the following journal characteristics to assess their potential relation to total RQS score: 1) impact factor (JIF) quartile; 2) citation index (JCI) quartile; 3) publication year; 4) JIF; 5) clinical or imaging journal domain ( “clinical” or “imaging” are attributed by using Web of Science, as described in [19]). JIF and JCI quartiles were obtained from Web of Science.

### Statistical analysis

All the analyses were performed using the RQS percentage score obtained by the most experienced rater. The main analysis evaluated the relationship between the study quality and journal features (quartile JIF, quartile JCI, publication years, JIF, clinical or imaging journal category). The skewed data distribution was assessed with the Kolmogorov–Smirnov test. To compare variables with a not- normal distribution, a Mann Whitney test was performed. Relationships between continuous variables were examined using Spearman correlation (ρ) for parametric variables with a not-normal distribution. Continuous variables are presented as median and interquartile range (IQR), categorical ones as count and percentage. All statistical analyses were performed using SPSS (SPSS version 27; SPSS, Chicago, IL). A *p* value < 0.05 was considered statistically significant.

## Results

### Literature review

In total, 289 articles were obtained from the initial search, of which 119 were duplicates. Of the remaining 170, 151 were rejected based on the selection criteria. Finally, 19 articles were included in the systematic review. The described flowchart is represented in Fig. 1 and included articles are summarized in Table 2.

**Table 2:** Characteristics of included articles

First Author	Journal	Year	Impact Factor	Quartile JIF	Quartile JCI	Journal main topic
Hassan Bagher-Ebadian[36]	Medical Physics	2020	4.071	Q1	Q1	radiology
Marta Bogowicz[53]	Radiation Oncology	2017	2.862	Q2	Q3	radiology
Marta Bogowicz[54]	Scientific Reports	2020	4.379	Q1	Q1	multidisciplinary sciences
Paula Bos MS[40]	Head & Neck	2020	3.147	Q1	NA	otolaryngology
K. Buch[37]	American Journal Of Neuroradiology	2015	3.124	Q1	NA	radiology
Y. Choi[39]	American Journal Of Neuroradiology	2020	3.825	Q2	Q2	radiology
Hesham Elhalawani[22]	Frontiers In Oncology	2018	4.137	Q2	Q2	oncology
Noriyuki Fujima[55]	European Journal Of Radiology	2020	3.528	Q2	Q1	radiology
Stefan P. Haider[23]	European Journal Of Nuclear Medicine And Molecular Imaging	2020	9.236	Q1	Q1	radiology
Daniel M. Lang[24]	Cancers	2021	6.639	Q1	Q1	oncology
Ralph TH Leijenaar[38]	British Journal Of Radiology	2018	1.939	Q3	Q3	radiology
Francesco Mungai[8]	Journal Of Computed Assisted Tomography	2017	1.385	Q2	NA	radiology
Sara Ranjbar[10]	La Radiologia Medica	2019	2.192	Q2	NA	radiology
Marco Ravanelli[42]	American Journal Of Neuroradiology	2018	3.256	Q2	Q2	radiology
Reza Reiazi[44]	Cancers	2021	6.639	Q1	Q1	oncology
Jiliang Ren[43]	European Radiology	2020	5.315	Q1	Q1	radiology
Beomseok Sohn[41]	Laryngoscope	2021	3.325	Q1	Q1	otolaryngology
Chong Hyun Suh[25]	Scientific Reports	2020	4.379	Q1	Q1	multidisciplinary sciences
Kaixan Yu[56]	Clinical And Translational Radiation Oncology	2017	3.124	Q2	NA	oncology

### RQS assessment

For both RQS total and percentage scores, ICC showed high agreement (89%; detailed information in supplementary materials). Supplementary tables S1-S3 report RQS item and total scores assigned by each rater to all the included articles. The quality of the included studies was very low (median score expressed as number 12) and RQS ranges from -2 to 15, corresponding to a median percentage score of 33 (14; 39) (Figure S1). Overall, 16 out of 19 (84%) authors included a well-documented imaging protocol, but no public protocol was used. In only 6 articles (31%) multiple segmentation by different physicians/algorithms/software were found in the radiomic pipeline. Lack of phantom study and imaging at multiple time points was observed in all studies. More than half authors performed feature reduction (13/19, 68%), while in 5 articles validation was completely missing. Although only 2 (10%) radiomic analyses were comprehensive of non-radiomic features, 13/19 (68%) demonstrated phenotypic differences, and all were compared with the current gold standard method. The main limitations, observed in the included articles, were the absence of re-application by a previously published cut off and the lack of calibration statistics, performed in only 10% (2 studies) and 5% (1 study), respectively. However, almost all authors (17/19, 89%) employed discrimination statistics. No researcher registered a prospective study in a trial database or performed a cost-effectiveness analysis, but 4 investigators did share the data obtained. Elhalawani shared the dataset generated for the study in Figshare repository [22], other authors share the code [23, 24], while Suh shared the datasets and the analysis on reasonable request [25].

### Subgroup analysis

**Fig. 2:**
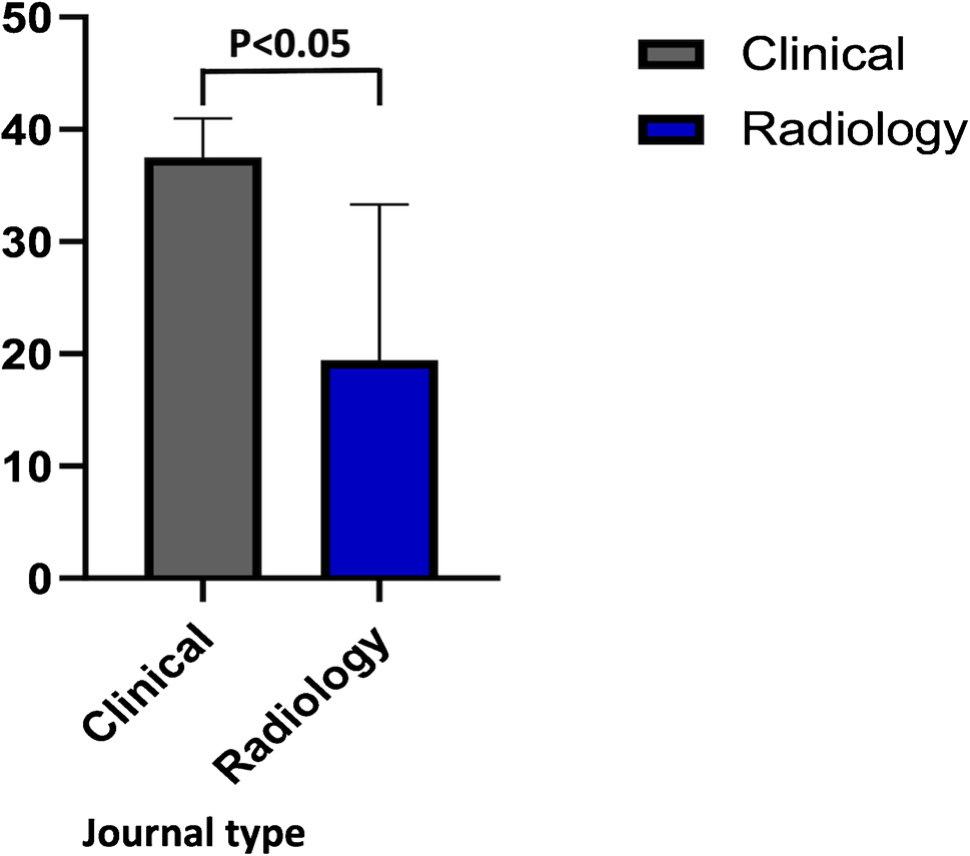
Distribution of the RQS in clinical and imaging journal

**Fig. 3:**
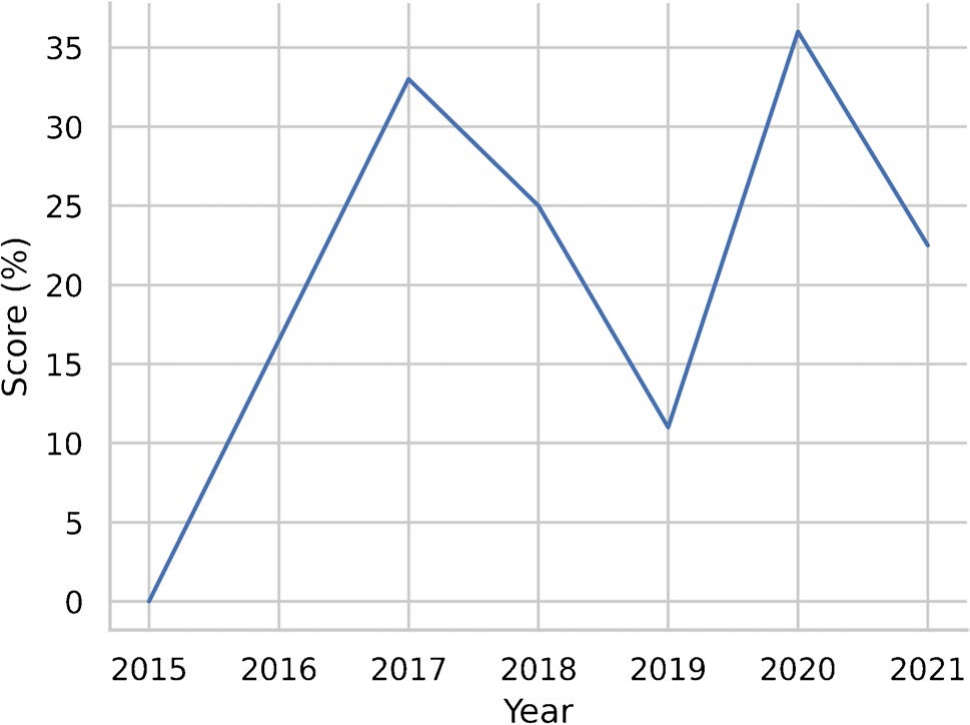
Distribution of median RQS% per year

**Fig. 4:**
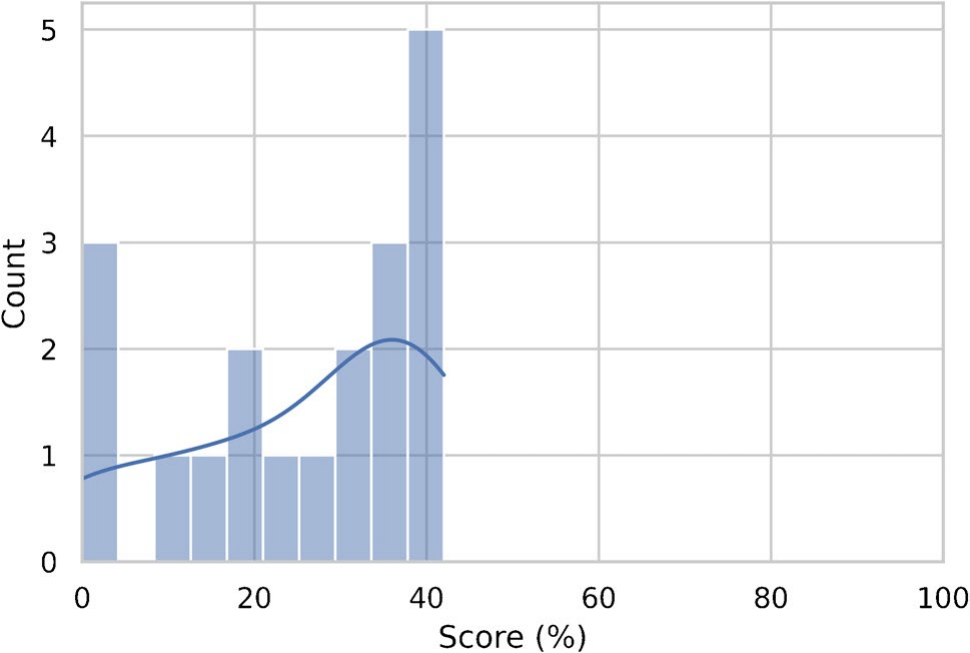
Normed histogram density distribution plot (bin value = 10) and kernel density plot of RQS% scores of the included articles

**Fig. 5:**
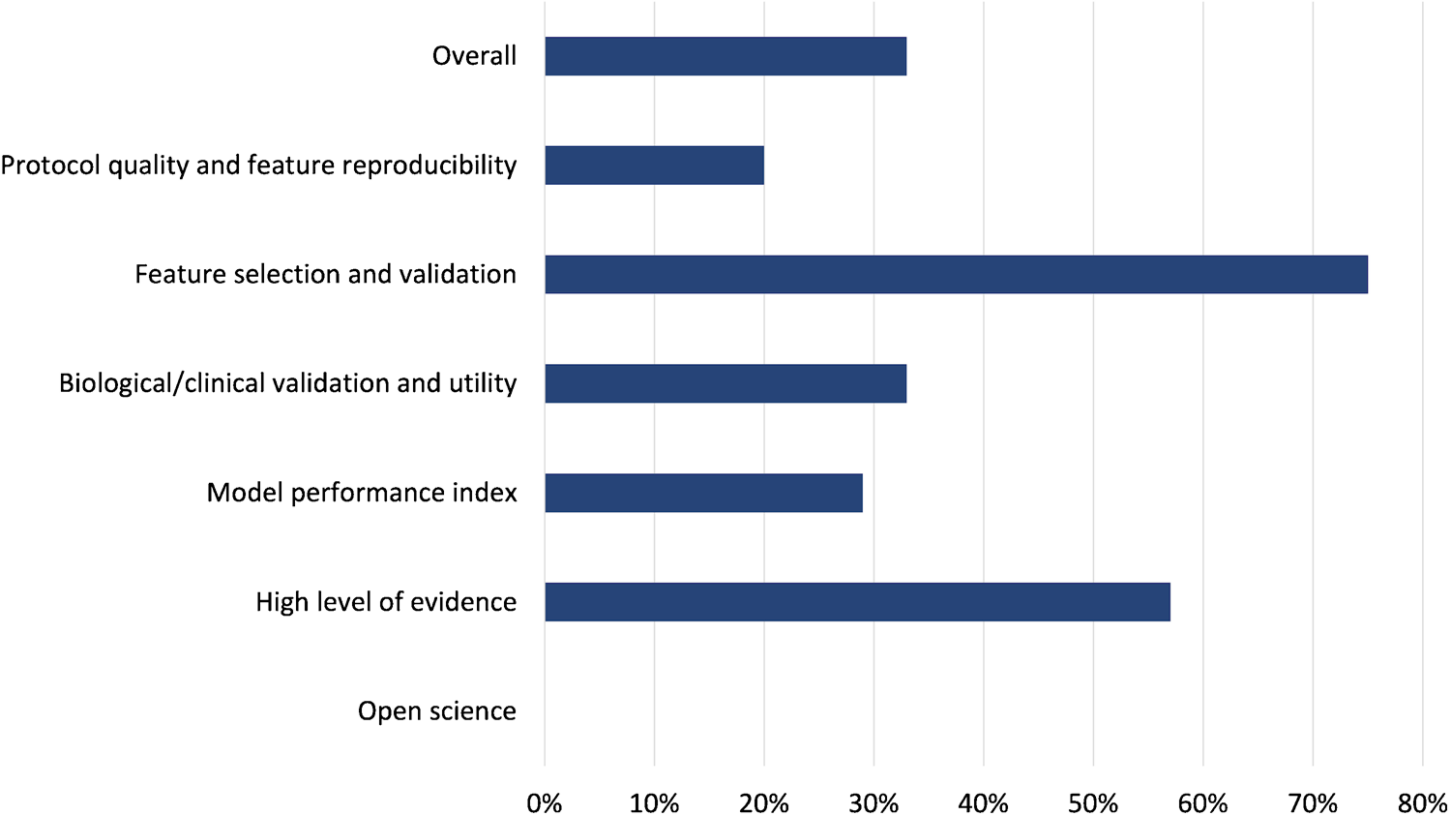
RQS% of the 19 studies according to the six key domains

Journal characteristics are summarized in Table 2, with 11/19 (57%) articles published on imaging journals. All had a wide variability of the quality indicators (mean IF 4.03 ± 1.86); half were published on high profile journals in their research field (10/19, 52% published on Q1 journals according to JIF; 9/19, 47% on Q1 journals by JCI). Most of papers were published in 2018 or later (15/19, 78%). More than half were based on CT images (13/19, 68%), 4/19 (21%) on MRI, and 2 studies on PET/CT (11%). In only 2 cases a deep learning (DL) analysis was carried out for OPSCC HPV status assessment. The results of the subgroup analysis according to the journal type demonstrated higher RQS score in articles published on clinical journals (Fig. 2). However, no correlations were observed for every single item score related to the journal type (clinical or radiological). Furthermore, no significant correlations were found between RQS score and JIF, quartile JIF/JCI or year of publication (details reported in supplementary material). In Figs. 3 and 4, we reported the distribution of the RQS expressed as percentage on the total of articles and the median RQS%. RQS% of the 19 studies according to the six key domains are reported in Fig. 5.

## Discussion

In the present systematic review, 19 radiomics and ML investigations published in the recent literature on the OPSCC were evaluated. In this setting, one of the most crucial issues in clinical practice is HPV status evaluation [26], and conventional imaging is not currently able to reliably replace the current gold standard (expression of p16 protein via immunohistochemistry from specimen [27]), despite various attempts [28–30]. The quality of included studies was very low (median score expressed as number 12, corresponding to a median percentage score of 33) with highest RQS equal to 15 (42%). Significantly higher RQS score was found in clinical journals compared to radiological ones, while no correlations were observed between RQS score and other journal characteristics (JIF, quartile JIF/JCI or year of publication).

Radiomics and texture analysis have tried to fill in the gaps in oral oncology and improve the performance of medical imaging. In the last years, several Authors attempted HPV status prediction radiomic modelling based on different imaging techniques, CT, MRI, or PET/CT. It is interesting to note that only a minority of papers employed DL, given the increasing attention to this approach [31]. Despite MRI having demonstrated its usefulness in HPV status assessment [32, 33], in our review most of the studies were focused on CT images. This could be due to some of its advantages: 1) wider availability in most hospitals [34]; 2) greater variability of MRI based on acquisition parameters as well as different scanners [35]. The resulting relevant radiomic features extracted from CT images have shown potential for HPV status prediction either in internal [8, 35–38] or external validation [39]. Other authors employed T1-weighted post contrast [25, 40, 41] and ADC [25, 42] images on MRI, and a combination of PET-based and CT-based radiomics on PET/CT [23]. In some cases, specific steps within the radiomic pipeline were also explored, such as comparison between 2 and 3D segmentation [43] and variations due to different CT scanners [44]. These are valuable as limitations in reproducibility of radiomics have been reported due to different CT reconstruction algorithms and image noise [45].

The RQS is one of the most known quality assessment checklists in the field of radiomics and was used to evaluate each paper’s strengths and weaknesses. Proposed by Lambin et al. [12], this score is composed by various items elaborated to reflect commonly employed steps in radiomic analysis pipelines, allowing quantitative and reproducible evaluation by peers. Although this score may be excessively strict when considering the practical issues of medical imaging research, it still represents a valuable and well-known tool [19]. Like other systematic reviews in other oncological imaging fields [20, 46, 47], the quality of included studies was very low and RQS ranged from -2 to 15, between 0 and 42% expressed as percentage. In line with the previous investigations [20, 46], some RQS items were satisfied to a greater extent than others. More than half of articles performed feature reduction, decreasing the risk of overfitting, and included non-radiomic features in a multivariable analysis. To demonstrate the utility of radiomics, all studies included a comparison to the current gold standard method, not always the case in other RQS systematic reviews. Some common missing steps were also recognised: less than 15% of radiomics pipelines comprised a cut-off analysis based on previously published reports, no cost-effectiveness analyses, phantom studies, or multiple time-point imaging were available. Open science implementation was also limited, with use of publicly available datasets practiced in very few cases, despite the advantages it might provide for testing reproducibility of proposed radiomics-based predictive models. Furthermore, in over a quarter of the included articles final model validation was entirely missing. Actual clinical implementation of these results will require more robust validation and possibly studies dedicated to this task.

Additional proxy quality indicators were included in our study. As journal quality indices, JIF and JIF quartiles were selected. The JIF is an index of relevance of the journal in its field of research, calculated from the citation average by year obtained by research published during the previous two years [48]. Other journal performance indicators, quartile ranking by JIF and JCI, were used. These provide additional insights, and JCI in particular reflects a 3-year citation window and is a field-normalized citation metric, unlike JIF [49]. Since Lambin proposed the total score expressed in percentage as quality assessment, the association between this and journal characteristics was evaluated. Interestingly, a significantly higher RQS was found in clinical journals compared to radiological ones. Probably, some RQS items, such as multivariable analysis with non-radiomics features and comparison to a gold standard, may benefit from the inclusion of a clinical researcher among the authors.

On the other hand, no significant correlation was found between RQS and either JIF and or JIF quartile. We also did not find a significant increase of RQS in relation to the year of publication. Similarly, the highest score was not associated to the better journals, in terms of JIF or JIF ranking. These results can again be interpreted in different ways: i) lack of uniformity in quality of radiomics/ML evaluation by reviewers, supported by the absence of association between excellence of the investigation and performance of the journal; ii) as hypothesized by an another systematic review [19], RQS items could not reflect journal or reviewer points of focus, such as patients selection criteria and the topic of the analysis; iii) the items proposed by Lambin [12] in the RQS may be too technical for general peer-review. The results of our analysis confirmed findings from previous reviews: not only the median RQS score in OPSCC articles was in line with that reported by authors [20, 50], but also the JIF was not related to quality of radiomic analysis [19]. However, our findings suggested that a higher RQS was found in clinical journals, contrary to what reported in our previous work [19] and Park et al. [50], although the latter described a not- significant trend for higher RQS in clinical journals [50].

The present review has some limitations. Firstly, the small sample size of included studies and their heterogeneity in terms of design and imaging modalities (MRI, CT, PET/CT). The inclusion of journal quality indicators, such as JIF, and JIF quartile and JCI, which are themselves influenced by potential biases [51], despite JIF being universally recognised as a valuable indicator [52]. As proposed by Lambin [12], the RQS should be expressed as a percentage score, but scores less than zero are all converted to 0%, losing the differences between all scores ranging from -8 to 0.

In conclusion, radiomics and ML studies for the prediction of HPV status in OPSCC have demonstrated low overall quality according to the RQS. While study quality was not related to journal quality, articles with best RQS scores were found in clinical journals. Future investigations in this field should take into account the issues highlighted in this review in order to improve upon previous experiences and facilitate a translation of promising research results to real-world clinical practice.

## Supplementary Information

Below is the link to the electronic supplementary material.

Supplementary file1 (DOCX 82 KB)

## Abbreviations

OPSCC: Oropharyngeal squamous cell carcinoma
HPV: Human papillomavirus
ML: Machine learning
RQS: Radiomic Quality Score
PRISMA: Preferred Reporting Items for Systematic reviews and Meta-Analyses
JIF: Journal impact factor
JCI: Journal citation index
ICC: Inter-reader intraclass correlation coefficient
DL: Deep learning
