# Supplementary material for: The impact of radiomics for human papillomavirus status prediction in oropharyngeal cancer: systematic review and radiomics quality score assessment
Source: Neuroradiology. 2022 Apr 23;64(8):1639–47. doi: 10.1007/s00234-022-02959-0 (PMC9271107; doi:10.1007/s00234-022-02959-0)
Supplement: Supplementary file 1 — Supplementary file1 (DOCX 82 KB) [file 234_2022_2959_MOESM1_ESM.docx]

**Detailed search strategy**

The full strings employed for the systematic literature search, formatted for the PubMed, Scopus and Web of Science search engine, respectively.

**Pubmed**

((((("machine learning"[MeSH Terms] OR ("machine"[All Fields] AND "learning"[All Fields])) OR "machine learning"[All Fields]) OR (("artificial intelligence"[MeSH Terms] OR ("artificial"[All Fields] AND "intelligence"[All Fields])) OR "artificial intelligence"[All Fields])) OR ("radiomic"[All Fields] OR "radiomics"[All Fields])) OR (((((((((("textural"[All Fields] OR "texturally"[All Fields]) OR "texture"[All Fields]) OR "texture s"[All Fields]) OR "textured"[All Fields]) OR "textures"[All Fields]) OR "texturing"[All Fields]) OR "texturization"[All Fields]) OR "texturize"[All Fields]) OR "texturized"[All Fields]) OR "texturizing"[All Fields])) AND (("human papillomavirus"[All Fields] OR "HPV"[All Fields]) OR "papillomavirus"[All Fields] OR "papilloma"[All Fields]) AND ("oropharyngeal"[All Fields] OR “oropharynx”[All Fields] OR “head and neck”[All Fields]) ("2000/01/01"[Date - Publication] : "2021/09/01"[Date - Publication]) AND "english"[Language]

**Scopus**

TITLE-ABS-KEY("machine learning" OR "artificial intelligence" OR "radiomics" OR "radiomic" OR "texture") AND TITLE-ABS-KEY("human papillomavirus" OR "HPV" OR "papillomavirus" OR "papilloma") AND TITLE-ABS-KEY("oropharyngeal" OR "oropharynx" OR "head and neck") AND PUBYEAR AFT 1999 AND LANGUAGE(english)

**Web of Science**

TOPIC: (machine learning OR artificial intelligence OR radiomics OR radiomic OR texture) AND TOPIC: (human papillomavirus OR HPV OR papillomavirus) AND TOPIC: (oropharyngeal OR oropharynx OR head and neck) - Timespan: 2000-2021.

**Table S1**. RQS perfomed by reviewer 1.

| **Criteria** | **Bagher-Ebadian_2020** | **Bogowicz_2017** | **Bogowicz_2020** | **Bos_2020** | **Buck_2015** | **Choi_2020** | **Elhalawani_2018** | **Fujima_2020** | **Haider_2020** | **Lang_2021** | **Lejenaar_2018** | **Mungai_2019** | **Ranjbar_2017** | **Ravanelli_2018** | **Reiazi_2021** | **Ren_2020** | **Sohn_2020** | **Suh_2020** | **Yu_2017** |
| --- | --- | --- | --- | --- | --- | --- | --- | --- | --- | --- | --- | --- | --- | --- | --- | --- | --- | --- | --- |
| **1** | 1 | 1 | 1 | 1 | 1 | 1 | 1 | 1 | 1 | 0 | 1 | 1 | 0 | 1 | 0 | 1 | 1 | 1 | 1 |
| **2** | 0 | 0 | 0 | 0 | 0 | 0 | 0 | 0 | 1 | 0 | 0 | 1 | 0 | 0 | 0 | 1 | 1 | 1 | 1 |
| **3** | 0 | 0 | 0 | 0 | 0 | 0 | 0 | 0 | 0 | 0 | 0 | 0 | 0 | 0 | 0 | 0 | 0 | 0 | 0 |
| **4** | 0 | 0 | 0 | 0 | 0 | 0 | 0 | 0 | 0 | 0 | 0 | 0 | 0 | 0 | 0 | 0 | 0 | 0 | 0 |
| **5** | 3 | 3 | 3 | 3 | -3 | 3 | 3 | -3 | 3 | -3 | -3 | -3 | 3 | -3 | 3 | 3 | 3 | 3 | 3 |
| **6** | 0 | 0 | 0 | 1 | 0 | 0 | 1 | 0 | 0 | 0 | 0 | 0 | 0 | 0 | 0 | 0 | 0 | 0 | 0 |
| **7** | 0 | 1 | 1 | 1 | 1 | 1 | 0 | 1 | 0 | 0 | 1 | 1 | 1 | 1 | 0 | 0 | 1 | 1 | 1 |
| **8** | 0 | 0 | 1 | 1 | 0 | 0 | 0 | 0 | 0 | 0 | 0 | 0 | 0 | 0 | 0 | 0 | 0 | 0 | 0 |
| **9** | 1 | 1 | 1 | 2 | 0 | 1 | 0 | 2 | 2 | 1 | 1 | 1 | 1 | 1 | 1 | 1 | 1 | 2 | 1 |
| **10** | 0 | 0 | 1 | 0 | 0 | 0 | 0 | 0 | 0 | 0 | 0 | 0 | 0 | 0 | 0 | 0 | 0 | 0 | 0 |
| **11** | 0 | 0 | 0 | 0 | 0 | 0 | 0 | 0 | 0 | 0 | 0 | 0 | 0 | 0 | 0 | 0 | 0 | 0 | 0 |
| **12** | 3 | 2 | 2 | 2 | -5 | 3 | 4 | 2 | 3 | 5 | 5 | -5 | -5 | -5 | 2 | -5 | 2 | 2 | 2 |
| **13** | 2 | 2 | 2 | 2 | 2 | 2 | 2 | 2 | 2 | 2 | 2 | 2 | 2 | 2 | 2 | 2 | 2 | 2 | 2 |
| **14** | 2 | 2 | 2 | 2 | 2 | 2 | 2 | 2 | 2 | 0 | 2 | 2 | 2 | 2 | 2 | 2 | 2 | 2 | 2 |
| **15** | 0 | 0 | 0 | 0 | 0 | 0 | 0 | 0 | 0 | 0 | 0 | 0 | 0 | 0 | 0 | 0 | 0 | 0 | 0 |
| **16** | 0 | 0 | 0 | 0 | 0 | 0 | 1 | 0 | 1 | 1 | 0 | 0 | 0 | 0 | 0 | 0 | 0 | 1 | 0 |
| **Total** | **12** | **12** | **14** | **15** | **-2** | **13** | **14** | **7** | **15** | **6** | **9** | **0** | **4** | **-1** | **10** | **5** | **13** | **15** | **13** |
| **%** | **33** | **33** | **39** | **42** | **0** | **36** | **39** | **19** | **42** | **17** | **25** | **0** | **11** | **0** | **28** | **14** | **36** | **42** | **36** |

**Table S2**. RQS perfomed by reviewer 2.

| **Criteria** | **Bagher-Ebadian_2020** | **Bogowicz_2017** | **Bogowicz_2020** | **Bos_2020** | **Buck_2015** | **Choi_2020** | **Elhalawani_2018** | **Fujima_2020** | **Haider_2020** | **Lang_2021** | **Lejenaar_2018** | **Mungai_2019** | **Ranjbar_2017** | **Ravanelli_2018** | **Reiazi_2021** | **Ren_2020** | **Sohn_2020** | **Suh_2020** | **Yu_2017** |
| --- | --- | --- | --- | --- | --- | --- | --- | --- | --- | --- | --- | --- | --- | --- | --- | --- | --- | --- | --- |
| **1** | 1 | 0 | 1 | 1 | 1 | 1 | 1 | 1 | 1 | 0 | 1 | 1 | 1 | 1 | 0 | 0 | 1 | 1 | 1 |
| **2** | 1 | 0 | 0 | 1 | 1 | 1 | 1 | 1 | 1 | 1 | 1 | 1 | 1 | 1 | 0 | 1 | 1 | 1 | 0 |
| **3** | 0 | 0 | 0 | 1 | 0 | 0 | 0 | 0 | 0 | 0 | 0 | 0 | 0 | 0 | 0 | 0 | 0 | 0 | 0 |
| **4** | 0 | 0 | 0 | 0 | 0 | 0 | 0 | 0 | 0 | 0 | 0 | 0 | 0 | 0 | 0 | 0 | 0 | 0 | 0 |
| **5** | 3 | 3 | 3 | 3 | -3 | 3 | 3 | -3 | 3 | -3 | -3 | -3 | 3 | -3 | 3 | 3 | 3 | 3 | 3 |
| **6** | 0 | 1 | 0 | 1 | 0 | 0 | 1 | 0 | 0 | 0 | 0 | 0 | 0 | 0 | 0 | 0 | 0 | 0 | 0 |
| **7** | 0 | 1 | 1 | 1 | 1 | 1 | 0 | 1 | 0 | 0 | 1 | 1 | 1 | 1 | 0 | 0 | 1 | 1 | 1 |
| **8** | 0 | 0 | 1 | 1 | 0 | 0 | 0 | 0 | 0 | 0 | 0 | 0 | 0 | 0 | 0 | 0 | 0 | 0 | 0 |
| **9** | 1 | 1 | 1 | 2 | 0 | 1 | 0 | 2 | 2 | 1 | 1 | 1 | 1 | 1 | 1 | 1 | 1 | 2 | 1 |
| **10** | 0 | 0 | 1 | 0 | 0 | 0 | 0 | 0 | 0 | 0 | 0 | 0 | 0 | 0 | 0 | 0 | 0 | 0 | 0 |
| **11** | 0 | 0 | 0 | 0 | 0 | 0 | 0 | 0 | 0 | 0 | 0 | 0 | 0 | 0 | 0 | 0 | 0 | 0 | 0 |
| **12** | 3 | 2 | 2 | -5 | -5 | 3 | 4 | 2 | 3 | 5 | 5 | -5 | -5 | -5 | 2 | -5 | 2 | 2 | 2 |
| **13** | 2 | 2 | 2 | 2 | 2 | 0 | 0 | 0 | 2 | 0 | 0 | 0 | 0 | 0 | 0 | 0 | 2 | 0 | 0 |
| **14** | 2 | 2 | 2 | 2 | 2 | 2 | 0 | 2 | 2 | 0 | 2 | 2 | 2 | 2 | 2 | 2 | 2 | 2 | 2 |
| **15** | 0 | 0 | 0 | 0 | 0 | 0 | 0 | 0 | 0 | 0 | 0 | 0 | 0 | 0 | 0 | 0 | 0 | 0 | 0 |
| **16** | 0 | 0 | 0 | 0 | 0 | 0 | 1 | 0 | 1 | 1 | 0 | 0 | 0 | 0 | 0 | 0 | 0 | 0 | 0 |
| **Total** | **13** | **12** | **14** | **10** | **-1** | **12** | **11** | **6** | **15** | **5** | **8** | **-2** | **4** | **-2** | **8** | **2** | **13** | **12** | **10** |
| **%** | **36** | **33** | **39** | **28** | **0** | **33** | **30** | **17** | **42** | **14** | **22** | **0** | **11** | **0** | **22** | **6** | **36** | **33** | **28** |

**Table S3**. RQS perfomed by reviewer 3.

| **Criteria** | **Bagher-Ebadian_2020** | **Bogowicz_2017** | **Bogowicz_2020** | **Bos_2020** | **Buck_2015** | **Choi_2020** | **Elhalawani_2018** | **Fujima_2020** | **Haider_2020** | **Lang_2021** | **Lejenaar_2018** | **Mungai_2019** | **Ranjbar_2017** | **Ravanelli_2018** | **Reiazi_2021** | **Ren_2020** | **Sohn_2020** | **Suh_2020** | **Yu_2017** |
| --- | --- | --- | --- | --- | --- | --- | --- | --- | --- | --- | --- | --- | --- | --- | --- | --- | --- | --- | --- |
| **1** | 1 | 1 | 0 | 1 | 1 | 1 | 1 | 1 | 2 | 1 | 0 | 1 | 0 | 1 | 1 | 0 | 1 | 1 | 0 |
| **2** | 1 | 1 | 1 | 1 | 1 | 1 | 1 | 1 | 1 | 0 | 1 | 1 | 1 | 0 | 0 | 1 | 1 | 1 | 1 |
| **3** | 0 | 0 | 0 | 0 | 0 | 0 | 0 | 0 | 0 | 0 | 0 | 0 | 0 | 0 | 0 | 0 | 0 | 0 | 0 |
| **4** | 0 | 0 | 0 | 0 | 0 | 0 | 0 | 0 | 1 | 0 | 0 | 0 | 0 | 0 | 0 | 0 | 0 | 0 | 0 |
| **5** | 3 | 3 | 3 | 3 | 3 | 3 | 3 | -3 | 3 | 0 | 3 | -3 | 3 | 0 | 3 | 3 | 3 | 3 | 3 |
| **6** | 0 | 1 | 0 | 1 | 0 | 1 | 1 | 0 | 1 | 0 | 0 | 0 | 0 | 1 | 0 | 0 | 0 | 0 | 0 |
| **7** | 1 | 1 | 0 | 1 | 0 | 1 | 0 | 0 | 1 | 0 | 1 | 0 | 1 | 0 | 0 | 0 | 0 | 0 | 0 |
| **8** | 1 | 1 | 1 | 1 | 0 | 0 | 0 | 0 | 1 | 0 | 0 | 0 | 0 | 0 | 0 | 0 | 1 | 1 | 0 |
| **9** | 2 | 2 | 1 | 2 | 1 | 2 | 2 | 2 | 2 | 1 | 0 | 2 | 2 | 1 | 2 | 1 | 2 | 2 | 2 |
| **10** | 0 | 0 | 1 | 2 | 1 | 0 | 0 | 0 | 0 | 0 | 0 | 2 | 2 | 1 | 2 | 1 | 2 | 2 | 2 |
| **11** | 0 | 0 | 0 | 0 | 0 | 0 | 0 | 0 | 0 | 0 | 0 | 0 | 0 | 0 | 0 | 0 | 0 | 0 | 0 |
| **12** | 3 | 2 | 2 | -5 | -5 | 3 | 4 | 2 | 3 | 5 | 5 | -5 | -5 | -5 | 2 | -5 | 2 | 2 | 2 |
| **13** | 0 | 2 | 0 | 2 | 0 | 2 | 0 | 0 | 0 | 1 | 0 | 0 | 0 | 0 | 0 | 0 | 0 | 0 | 0 |
| **14** | 2 | 2 | 2 | 2 | 2 | 2 | 2 | 2 | 2 | 0 | 2 | 2 | 2 | 2 | 2 | 2 | 2 | 2 | 2 |
| **15** | 0 | 0 | 0 | 0 | 0 | 0 | 0 | 0 | 0 | 0 | 0 | 0 | 0 | 0 | 0 | 0 | 0 | 0 | 0 |
| **16** | 0 | 0 | 0 | 0 | 0 | 0 | 1 | 1 | 1 | 1 | 0 | 1 | 0 | 0 | 1 | 0 | 1 | 2 | 1 |
| **Total** | **14** | **16** | **11** | **11** | **4** | **16** | **15** | **6** | **18** | **9** | **12** | **1** | **6** | **1** | **13** | **3** | **15** | **16** | **13** |
| **%** | **39** | **44** | **30** | **30** | **11** | **44** | **42** | **17** | **50** | **25** | **33** | **3** | **17** | **3** | **36** | **8** | **42** | **44** | **36** |

**Table S4**

RQS interclass correlation coefficient (ICC) results.

|  | **RQS** | **RQS%** |
| --- | --- | --- |
| b | 19 | 19 |
| Raters | 3 | 3 |
| Model | twoway | twoway |
| Type | agreement | agreement |
| Unit | Single | single |
| icc.name | ICC(A,1) | ICC(A,1) |
| Value | **0.89** | **0.89** |
| r0 | 0 | 0 |
| Fvalue | 42.05 | 42.08 |
| df1 | 18 | 18 |
| df2 | 8.96 | 9.96 |
| p.value | 0.00 | 0.00 |
| conf.level | 0.95 | 0.95 |
| Lbound | **0.66** | **0.69** |
| Ubound | **0.96** | **0.96** |

| **Tables S5a and S5b**  Distribution of RQS based on journal characteristics (quartile JIF and quartile JCI).   \|  \| Quartile JIF \| N \| Median \| IQR \| P value \| \| --- \| --- \| --- \| --- \| --- \| --- \| \| RQS \| Q1 \| 10 \| 34.72 \| 15.97-41.66 \| >0.05 \| \| Q2/Q3 \| 9 \| 25 \| 5.55-36.11 \| >0.05 \| | | | | | |
| --- | --- | --- | --- | --- | --- | --- | --- | --- | --- | --- | --- | --- | --- | --- | --- | --- | --- | --- | --- | --- | --- | --- |
|  | Quartile JCI | N | Median | IQR | P value |
| RQS | Q1 | 9 | 33.33 | 18.05-40.27 | > 0.05 |
|  | Q2/Q3 | 5 | 33.33 | 12.5-37.5 | > 0.05 |

**Table S6**

Distribution of RQS% based on the year of publication (before and after 2020).

|  | Data | N | Median | IQR | P value |
| --- | --- | --- | --- | --- | --- |
| RQS | Before 2020 | 8 | 18 | 0-35.41 | >0.05 |
|  | After 2020 | 11 | 36.11 | 19.44-41.66 | >0.05 |

**Table S7**

Spearman correlation between RQS% and Impact factor of the year of publication.

|  | | RQS | IF |
| --- | --- | --- | --- |
| RQS | Spearman correlation | 1 | .396 |
|  | Sign. (two tails) |  | .093 |
|  | N | 19 | 19 |
| IF | Spearman correlation | .396 | 1 |
|  | Sign. (two tails) | .093 |  |
|  | N | 19 | 19 |

**Table S8**

Mann-Whitney test results for testing the relationship between each RQS item and the journal type (clinical and radiological).

| **RQS Item** | **Median (IQR)** | **p-value** |
| --- | --- | --- |
| Item 1 | 1 (0) | 0.60 |
| Item 2 | 0 (1) | 0.72 |
| Item 3 | 0 (0) | 1.00 |
| Item 4 | 0 (0) | 1.00 |
| Item 5 | 3 (6) | 0.24 |
| Item 6 | 0 (0) | 0.40 |
| Item 7 | 1 (1) | 0.72 |
| Item 8 | 0 (0) | 0.40 |
| Item 9 | 1 (0) | 0.90 |
| Item 10 | 0 (0) | 0.66 |
| Item 11 | 0 (0) | 1.00 |
| Item 12 | 2 (8) | 0.31 |
| Item 13 | 2 (0) | 1.00 |
| Item 14 | 2 (0) | 0.66 |
| Item 15 | 0 (0) | 1.00 |
| Item 16 | 0 (0) | 0.31 |
| Total | 12 (9) | **0.02** |

**Figure S1**

RQS total scores for the included articles.

**
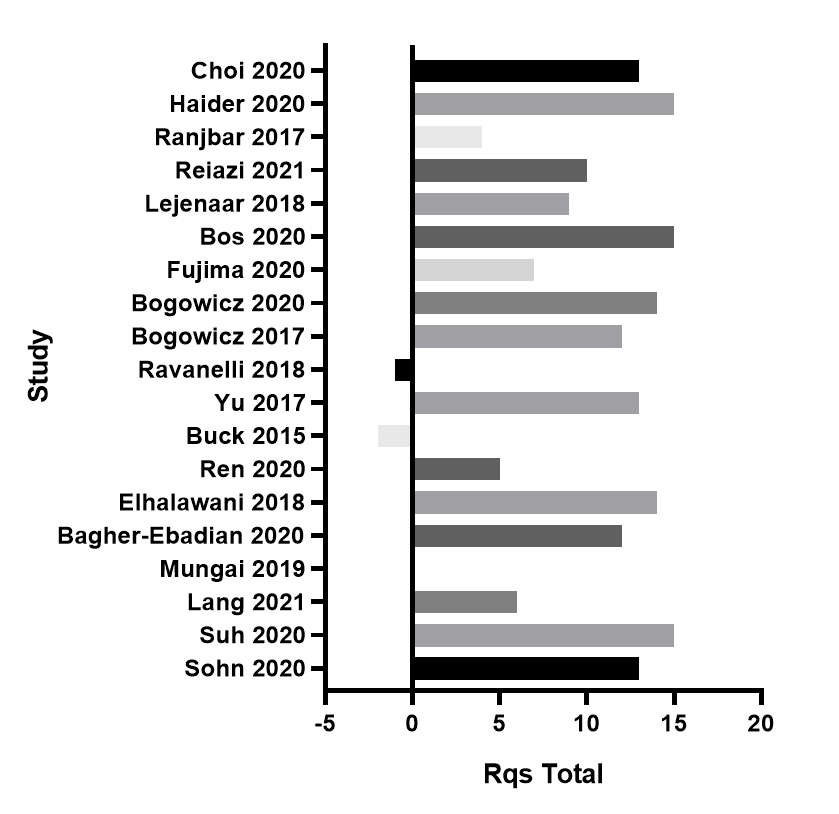
**
